# Removal from the plasma of the free and esterified forms of cholesterol and transfer of lipids to HDL in type 2 diabetes mellitus patients

**DOI:** 10.1186/1476-511X-11-65

**Published:** 2012-06-07

**Authors:** Carolina P Oliveira, Raul C Maranhão, Marina P Bertato, Bernardo L Wajchenberg, Antonio C Lerario

**Affiliations:** 1 Heart Institute (InCor) of the Medical School Hospital, University of Sao Paulo, São Paulo, Brazil; 2 Endocrinology Service of the Medical School Hospital, University of Sao Paulo, São Paulo, Brazil; 3 Faculty of Pharmaceutic Sciences, University of Sao Paulo, São Paulo, Brazil; 4 Instituto do Coração do HC-FMUSP, Av Dr. Enéas de Carvalho Aguiar, 44, CEP- 05423-000, São Paulo, SP, Brazil

**Keywords:** Type 2 diabetes mellitus, Lipoprotein, Low density lipoprotein, High density lipoprotein, Lipid transfer, Nanoparticles, Emulsion

## Abstract

**Background:**

The aim was to investigate new markers for type 2 diabetes (T2DM) dyslipidemia related with LDL and HDL metabolism. Removal from plasma of free and esterified cholesterol transported in LDL and the transfer of lipids to HDL are important aspects of the lipoprotein intravascular metabolism. The plasma kinetics (fractional clearance rate, FCR) and transfers of lipids to HDL were explored in T2DM patients and controls, using as tool a nanoemulsion that mimics LDL lipid structure (LDE).

**Results:**

^14^C- cholesteryl ester FCR of the nanoemulsion was greater in T2DM than in controls (0.07 ± 0.02 vs. 0.05 ± 0.01 h^-1^, p = 0.02) indicating that LDE was removed faster, but FCR ^3^ H- cholesterol was equal in both groups. Esterification rates of LDE free-cholesterol were equal. Cholesteryl ester and triglyceride transfer from LDE to HDL was greater in T2DM (4.2 ± 0.8 vs. 3.5 ± 0.7%, p = 0.03 and 6.8 ± 1.6% vs. 5.0 ± 1.1, p = 0.03, respectively). Phospholipid and free cholesterol transfers were not different.

**Conclusions:**

The kinetics of free and esterified cholesterol tended to be independent in T2DM patients and the lipid transfers to HDL were also disturbed. These novel findings may be related with pathophysiological mechanisms of diabetic macrovascular disease.

## Background

Dyslipidemia of type 2 diabetes mellitus (T2DM) is consequent to alterations in key processes of the plasma lipid metabolism modulated by insulin, such as lipolysis by lipoprotein lipase and hormone-sensitive lipase [1-3]. The function of cell receptors that remove lipoproteins from the plasma is also influenced by insulin [4-6]. T2DM dyslipidemias characterized by accumulation of triglyceride-rich lipoproteins, such as VLDL, as well as low HDL cholesterol. Diabetic dyslipidemia predisposes to development of cardiovascular disease [7-10], the major cause of mortality of those patients [9,11,12].

LDL cholesterol concentration is not typically increased in T2DM but LDL is subjected to important changes in this disease, such as increase in the small, dense LDL subclass, that is considered the most atherogenic [13-15]. Like the other lipoprotein classes, LDL contains both forms of cholesterol, the free and the esterified form [2,3]. Free cholesterol is located in the surface layer of the lipoprotein particles and can easily diffuse in the surrounding aqueous medium. Cholesteryl esters are located in the lipoprotein core wherein they are more stable than the free form [2,16,17]. In the circulation, free cholesterol is esterified by the action of lecithin cholesterol acyl transferase (LCAT), using apolipoprotein (apo) A1 as a co-factor [2,3]. This process occurs mainly in HDL, because most of the apo A1 pool is found in this fraction [16,17]. HDL receives free cholesterol, phospholipids and other lipids from the other lipoprotein classes and from cells of peripheral tissues. Transfer of free cholesterol from cells to HDL is mediated by ATP-binding cassette transport 1 (ABCA1) system.

The process of lipid transfers among lipoprotein classes is mediated by transfer proteins, such as cholesteryl ester transfer protein (CETP) and phospholipid transfer protein (PLTP) [2,3]. Lipid transfers are essential parts of cholesterol reverse transport and are particularly important in HDL metabolism. This lipoprotein is formed into the intravascular compartment by lipidation of apo A1, originating disks that, with further acquisition of lipids and cholesterol esterification, changes into round-shaped particles [2,3], so that HDL is constantly being remodeled. As presence of T2DM may change the activity of the transfer proteins [18,19], the lipid transfers to HDL and the HDL metabolism may also be affected [20].

In previous studies [21-27], we explored the intravascular metabolism of free and esterified cholesterol carried in an artificial nanoemulsion (LDE) that mimics the lipidic structure of LDL but does not contain protein. In this approach, LDE is doubly labeled with ^14^ C-cholesteryl esters and ^3^ H-cholesterol and injected intravenously in a bolus; blood is sequentially sampled for determination of the plasma kinetics of both labels. In contact with plasma, LDE acquires exchangeable apolipoproteins such as apo E [21,22] that endows the nanoemulsion to bind to LDL receptors [23,25]. The validity of the LDE method to test the LDL metabolism in the clinical setting was shown in several studies [21-27].

LDL metabolism can be altered even in presence of normal LDL cholesterol levels. Increase in small dense LDL subfraction is exemplary for this situation, and it has been shown the benefits of cholesterol lowering by statin treatment administered to normocholesterolemic T2DM patients [28,29]. In this setting, the current study aimed to investigate whether the kinetics in the plasma of free and esterified cholesterol and the lipid transfers to HDL are altered in T2DM patients with normal LDL cholesterol. LDE labeled with ^14^ C-cholesteryl esters and ^3^ H-cholesterol was used to probe this metabolism in cases versus control subjects. LDE was also used as lipid donor to HDL in an *in vitro* assay [30] to test in both groups the simultaneous transfer of free and esterified cholesterol, phospholipids and triglycerides to HDL.

## Results

As shown in Table 1, the glycemic control of the participant T2DM patients was not good, as judged by their HbA_1c_ levels. The concentration of total serum cholesterol and total serum free cholesterol, as well as the LDL cholesterol concentration, were similar in T2DM and controls. HDL cholesterol tended to be lower in T2DM patients, although this difference did not attain statistical significance (p = 0.06). Serum triglyceride concentration was higher in T2DM (p = 0.05). Apo A1, apo B and apo E did not differ between the two groups.

**Table 1:** Serum biochemical parameters of the the Type 2 Diabetes Mellitus (T2DM) and Control groups

	**T2DM**	**Control**	***P***
	**(n = 15)**	**(n = 11)**	
Fasting plasma glucose (mmol/L)	9.4 ± 5.3	4.6 ± 0.4	<0.01
HbA_1c_ (%)	8.9 ± 1.9	5.6 ± 0.4	<0.01
Cholesterol (mmol/L)	4.7 ± 1.0	4.9 ± 0.9	0.61
Non-HDL	3.6 ± 0.9	3.4 ± 0.8	0.59
LDL	2.9 ± 0.4	2.8 ± 0.7	0. 60
HDL	1.1 ± 0.3	1.5 ± 0.6	0.06
Triglycerides (mmol/L)	2.1 ± 1.3	1.3 ± 0.6	0.05
Free Cholesterol (mmol/L)	1.5 ± 0.3	1.5 ± 0.4	0.96
Apolipoproteins (g/L)			
A1	1.34 ± 0.21	1.53 ± 0.40	0.19
B	0.95 ± 0.25	0.87 ± 0.20	0.38
E	0.05 ± 0.02	0.04 ± 0.01	0.29

Data are means ± SD.

**Figure 1:**
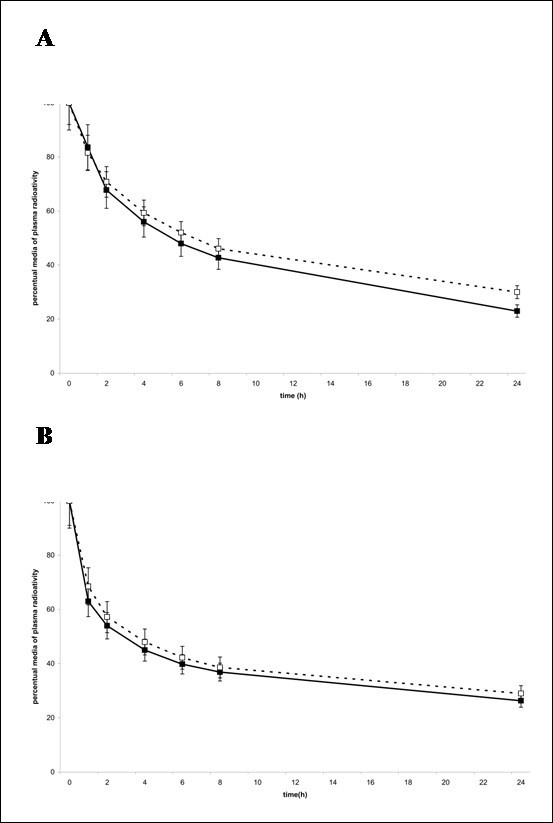
**Plasma decay curve of**^**14**^ **C-cholesteryl ester (A) and**^**3**^ **H-cholesterol (B) obtained from type 2 diabetes mellitus (black square) and control (white square) groups.** The doubly labeled nanoemulsion was intravenously injected in a bolus, and blood samples were drawn in pre-established intervals over 24 h for measurement of the radioactivity in a scintillation solution. Data are expressed as mean ± SD

The plasma decay curves of both nanoemulsion labels are shown in Figure 1. While the free cholesterol curves of T2DM patients and controls were similar, the cholesteryl ester curve seems faster in the patients than in controls.

Indeed, the removal from the plasma of the nanoemulsion cholesteryl esters was faster in T2DM than in controls (Figure 1), as confirmed by their greater FCR of this label (p = 0.02, Table 2). As expected from inspection of the curves in Figure 1, the two groups showed similar FCR of the free cholesterol (p = 0.75).

**Table 2:** Fractional Clearance Rates and kinetic parameters of LDE radioactive lipid labels in the Type 2 Diabetes Mellitus (T2DM) and Control groups.

	**T2DM**	**Control**	***P***
	**(n = 15)**	**(n = 11)**	
FCR ^14^ C-CE (h^-1^)	0.07 ± 0.02	0.05 ± 0.01	0.02
k _1.0_^14^ C-CE	0.30 ± 0.15	0.30 ± 0.26	0.91
k _1.2_^14^ C-CE	0.90 ± 1.20	0.97 ± 1.44	0.44
k _2.0_^14^ C-CE	0.05 ± 0.02	0.03 ± 0.01	0.02
FCR ^3^ H-C(h^-1^)	0.05 ± 0.02	0.05 ± 0.02	0.75
k _1.0_^3^ H-C	0.88 ± 0.83	0.86 ± 0.63	0.88
k _1.2_^3^ H-C	0.89 ± 1.02	0.90 ± 1.02	1.00
k _2.0_^3^ H-C	0.02 ± 0.01	0.03 ± 0.02	1.00

Data are express as mean ± SD. LDE, non-protein LDL-like nanoemulsion. ^14^ C- CE, ^14^ C-cholesteryl ester. ^3^ H-C, ^3^ H-cholesterol.

Table 3 shows the percentual of transfer from the donor nanoemulsion lipids to the HDL fraction after 1 h incubation of the nanoemulsion with whole plasma. Patients with T2DM showed increased transfer of the core lipids, namely cholesteryl esters (p = 0.03) and triglycerides (p = 0.03), to HDL than the controls. In contrast, the transfer of the surface lipids, phospholipids and free cholesterol, were similar.

**Table 3:** **Lipid transfer*in vitro*from LDE to HDL particles of the Type 2 Diabetes Mellitus (T2DM) and Control groups**

	**T2DM**	**Control**	***P***
	**(n = 15)**	**(n = 11)**	
^3^ H- cholesteryl ester (%)	4.2 ± 0.8	3.5 ± 0.7	0.03
^14^ C-phosphatidylcholine (%)	24.1 ± 2.7	22.0 ± 0.9	0.15
^3^ H- triglycerides (%)	6.8 ± 1.6	5.0 ± 1.1	0.03
^14^ C- cholesterol (%)	9.2 ± 3.0	7.5 ± 2.6	0.23

Data are expressed as mean ± SD. The value indicated is the percentage of the radioactivity of each lipid component in the nanoemulsion that was transferred to the plasma HDL fraction after 1 h incubation.

## Discussion

T2DM patients who were on metformin treatment alone or with additional sulphonylurea or insulin, showed in average poor glycemic control together with mild to high-triglycerides and borderline low HDL cholesterol. This conforms to a typical T2DM dyslipidemia.

In the T2DM patients, LDL cholesterol was normal and did not differ from the values from the control subjects. In those patients, the removal from the plasma of the cholesteryl ester component of LDE was faster than that measured in the control subjects. As the cholesteryl ester FCR closely depicts the removal from the plasma of the LDE particles, it can be assumed that inT2DM patients LDE was cleared more efficiently than control subjects. A plausible interpretation for this result resides on that all the study patients were on use of metformin. The mechanism of action of this drug is related at least partially with activation of AMP- activated protein kinase (AMPK) [31]. It has been shown that AMPK decreases hydroxyl-methilglutaryl- CoA reductase activity, leading to upregulation of LDL receptors [32,33]. Because, similarly to native LDL [34], LDE is removed by LDL receptors [21,24,25], it is conceivable that metformin use elicited the accelerated LDE cholesteryl ester clearance observed in T2DM.

In contrast, it is noteworthy that the free cholesteryl component of LDE was removed at similar rates in T2DM and in controls. This could suggest that the free form of cholesterol is released from the LDE particles while in the circulation [24,26], possibly being transferred to the native lipoprotein classes [30] that are removed slower than LDE [22]. Another possibility stands on the selective uptake of cholesteryl esters by cells of liver and macrophages [35,36], that could accelerate the removal from the plasma of those components without changing the free cholesterol removal in T2DM patients. In lipoprotein structure, free cholesterol, which is located in the particle surface, is more unstable than cholesteryl esters which is placed in the particle core. The esterified form depends on the transfer proteins to exit the lipoprotein but free cholesterol may diffuse into the surrounding aqueous media and thereafter may be incorporated into other lipoproteins or cell membranes.

Tracing of the lipid components of native LDL in the circulation of subjects is not practical, due to the difficulties of the labeling technique, which makes the nanoemulsion approach particularly useful. Thus, to our knowledge, this is the first study in which the kinetics of free and esterified cholesterol were explored in diabetes. In studies in which the clearance of native LDL labeled in the apo B component was determined, LDL clearance has been described as greater, equal or smaller [37-40] than in controls. Those contradictory results can be ascribed to many intervening factors, such as different treatments or plasma lipid profiles of the patients.

The important aspect of HDL metabolism investigated here, the process of lipid transfers to the lipoprotein, is determinant of HDL composition and metabolism. Thus, it is likely that atheroprotective functions of the lipoprotein may also be affected by lipid transfers, such as the antioxidant, anti-inflammatory, antithrombotic, and vasodilator actions [20]. In previous studies, we showed that HDL transfers were altered in precocious coronary artery disease [41] and in patients with heart grafts [42], a condition that is associated with development of heart graft coronary disease, an accelerated atherosclerosis process. In the current study, T2DM patients showed increased transfers of triglycerides and cholesteryl esters that make-up the lipoprotein core, whereas the transfer of phospholipids and free cholesterol that are the constituents of lipoprotein surface was not affected [41,42]. This finding is probably related with the increase in action of CETP that may occur in T2DM [16,17] and facilitating the transfer of core lipids. Enrichment of HDL with triglycerides is related with loss of function and stability of the lipoprotein [1,16,17]. This has been ascribed to the interactions of HDL with hepatic lipase that lead to acceleration of the lipoprotein clearance and reduction of HDL plasma levels [17]. In fact, it was observed here a trend for lower HDL–cholesterol in the T1DM group. In a previous study, we compared lipid transfers to HDL measured in T2DM without microalbuminuria with those with microalbuminuria, that is a more advanced disease stage and no difference was found between the two groups. This may suggest that aggravation of diabetes complications does not interfere with the lipid transfers [43].

As a limitation of this study, non-protein LDE has differences in chemical composition regarding LDL, and also differs in binding to LDL receptors by recognition of apo E rather than apo B [22], as in the native lipoprotein [34]. Nonetheless, as shown in several studies [21,24,26,27], by using LDE as probe for the LDL metabolic pathway in case–control protocols it is possible to detect alterations in diseased states and under the effect of drugs [24] that could be fairly extrapolated to native LDL metabolism. As the groups of T1DM and controls had similar apo E plasma concentrations, it can be assumed that apo E adsorption to LDE particles did not influence FCR of the nanoemulsion.

## Conclusion

The independent removal from the plasma of the free and of the esterified forms of cholesterol despite normal LDL cholesterol levels and the alterations in the lipid transfers to HDL found in T2DM suggest the existence of novel pathophysiologic pathways that can eventually be related with diabetic macrovascular disease.

## Methods

### Study subjects

The participants in the study were selected from the Outpatient Clinics of the Endocrinology Section of the Hospital of the University of São Paulo Medical School. The inclusion criteria were men and postmenopausal women, with 40 to 70 years of age without previous history of cardiovascular disease. Ten out of fifteen of the T2DM patients and five out of eleven of the control subjects presented systemic arterial hypertension and were on antihypertensive drugs and well controlled with a maximum systolic blood pressure of 130 mmHg and diastolic of 85 mmHg. Regarding the lipid levels total cholesterol was ≤ 6 mmol/L and LDL ≤ 4 mmol/L. The exclusion criteria were the use of medications that have significant action on lipid metabolism such as statins, fibrates, glucocorticoids and thiazolidinediones, presence of nephropathy (the presence of microalbuminuria, as indicated to urinary ratio microalbuminuria/creatinine <30 μg/mg and serum creatinine below 98 μmol/L in women and 115 μmol/L in men), proliferative retinopathy and neuropathy. Furthemore, the presence of chronic diseases such as heart failure, chronic obstructive pulmonary disease, inflammatory disease and cancer were also exclusion factors. T2DM patients had the duration of the disease less than 15 years. The characteristics of the study patients are depicted in Table 4. T2DM diabetes mellitus patients had mean known duration of disease 8 ± 4.0 years. There were no differences between both groups regarding age and sex distributions, but the patients had higher BMI (p < 0.01) and waist circumference (p < 0.05) than controls. All were being treated with metformin, but 8 were also treated with sulfonylurea (glyburide), 2 also with insulin and 2 with additional insulin and sulphonylurea.

**Table 4:** Physical characteristics and current medications of the Type 2 Diabetes Mellitus (T2DM) and Control groups

	**T2DM**	**Control**	***P***
N	15	11	
Age (years)	58.9 ± 4.8	54.6 ± 5.5	0.06
Sex (M/F)	7/8	5/6	1.00
Weight (kg)	82.1 ± 11.7	74.9 ± 7.3	0.08
BMI (kg/m^2^)	31.9 ± 4.6	27.1 ± 2.4	<0.01
Waist Circumference (cm)	104.9 ± 9.8	94.2 ± 7.3	<0.01
Arterial Hypertension	10	5	0.43
Familial history CVD	5	5	0.69
Current smoking	3	2	0.22
Current medications			
Metformin	15	-	-
Sulfonylurea	8	-	-
Insulin	4	-	-
ACEi	9	4	0.39
ARB	1	1	1.00
Ca channel blockers	3	2	0.49
Thiazides	2	1	1.00
AAS	4	0	0.10

Data are means ± SD. CVD, cardiovascular disease. ACEi, angiotensin-converting enzyme inhibitor. ARB, angiotensin II receptor blocker. Ca, calcium.

### Serum biochemical analysis

Blood samples for determination of laboratorial parameters were collected after 12 h fast on the same day the kinetic study were preformed. Commercial enzymatic colorimetric methods were used for the determination of total cholesterol, triglycerides and HDL cholesterol. LDL cholesterol was calculated by a direct method (Roche Diagnostics, Mannheim, Germany). Plasma apo A1 and apo B were assayed by turbidimetry (Roche Diagnostics) and apo E by nephelometry (Roche Diagnostics), Glyco Hemoglobin (HbA_1c_) was measured by HPLC (National Glyco Hemoglobin Standardization Program - NGSP-USA, considering normal range 4,1 a 6%).

### LDE preparation

LDE was prepared from a lipid mixture composed of 40 mg cholesteryl oleate, 20 mg egg phosphaditylcholine, 1 mg triolein and 0.5 mg of cholesterol purchased from Sigma Chemical (St. Louis, MO). ^14^ C- cholesteryl oleate and ^3^ H- cholesterol purchased from PerkinElmer (Waltham, MA) were added to the mixture. Emulsification of lipids by prolonged ultrasonic irradiation in aqueous media and the procedure of two-step ultracentrifugation of the crude emulsion with density adjustment by addition of KBr to obtain LDE was carried out as described by Maranhão et al. [22]. The final lipidcomposition of LDE was 64% phospholipids, 33% cholesteryl oleate, 2% triacylglycerols, and 1% cholesterol [20]. LDE was dialyzed against a saline solution and sterilized by passage through 0.22-μm filter for injection into the patients. The entire LDE preparation procedure was performed in a laminar flux. All glassware used in this study was made pyrogen free by exposure to dried steam at 180°C for 2hand sterilized by wet steam at 120°C for 30 min. All plastic materials were sterilized by exposure to ultraviolet light.

### Nanoemulsion plasma kinetics

All the patients were asked to arrive at the laboratory by 7 a.m. after a 12 h fasting. The basal collection of blood samples was done for the blood assays, as explained previously. LDE labeled with^14^C-cholesteryl oleate (37 kBq) and ^3^ H-cholesterol (74 kBq), in a total 5–6 mg in a volume 500 μL was intravenously injected in a bolus. Blood samples were taken over 24 h (5’, 1 h, 2 h, 4 h, 6 h, 8 h and 24 h). Participants were allowed to eat low-fat meals, on the evening before the test day and after the first blood collection and at about 1:00 p.m., since it is known that low-fat meals do not interfere with plasma removal of the LDE [21]. Plasma samples were separated by centrifugation and 1 mL was transferred to counting vials containing 5 mL scintillation solution, and counted in a scintillation counter (1600 TR model, Hewlett-Packard, Palo Alto, CA).

### Estimation of fractional clearance rate of the radioisotopes

**Figure 2:**
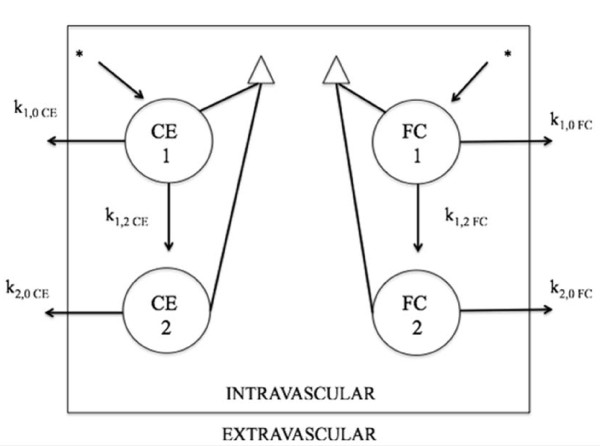
**Compartmental model used for analysing LDE**^**14**^ **C- cholesteryl ester (**^**14**^ **C-CE) and**^**3**^ **H-cholesterol curves (**^**3**^ **H-FC).** The model consists of four discrete compartments: two for ^14^ C-CE and two for ^3^ H-FC labels. All compartments are in the intravascular space (1_CE_, 2_CE_, 1_FC_ and 2 _FC_). LDE, a non-protein lipoprotein nanoemulsion labeled with ^14^ C-CE and ^3^ H-FC were injected intravenously in a bolus (arrow with asterisk) into compartment 1_CE_ and 1_FC_, respectively. A fraction k_1,0CE_ and k_1,0 FC_ of the labeled lipids is removed to the extravascular space. Competitively, fraction k_1,2CE_ and k_1,2FC_ of the injected lipids are converted into compartments 2_CE_ and 2_FC_ due to the incorporation of apolipoproteins available in the plasma. Subsequently, the material of those compartments are transferred to the extravascular space following the k_2,0CE_ and k_2,0FC_ routes. The samplings, represented by triangles correspond to the indiscriminate combination of compartments 1 and 2.

Fractional clearance rate (FCR) of ^14^ C-cholesteryl ester and ^3^ H-free cholesterol contained in LDE was calculated according to the method described elsewhere [27] e Silva et al. 2011 [44], defined by the sum of two exponential functions, obtained from the remaining radioactivity found in plasma after injection, as y = (a_1_.e^-b^_1_^t^ + a_2_.e^-b^_2_^t^), where y is the curve the radioactivity plasma decay in function of time (t); *a* indicates the linear coefficient and *b* the angular coefficient, which represents the FCR in hours^-1^ (h^-1^). The model consists of two discrete pools, one intravascular pool in dynamic equilibrium with an extravascular pool, assuming that all input or exit of the radiolabeled lipid occurs from the intravascular pool. The fractional clearance rate of the radiolabeled lipid was estimated as FCR = (a_1_/b_1_ + a_2_/b_2_)^-1^, which is essentially the inverse of the area under the activity-time curve. Calculations were performed using the ANACOMP computer software [45]. The compartmental model is illustrated in Figure 2.

### Lipid transfer from LDE to HDL *in vitro*

The transfer rates of cholesteryl ester, phospholipid, free cholesterol, and triglyceride from LDE to HDL were measured according to Lo Prete et al. [30]. Plasma with EDTA in a volume of 200 μL was incubated with 50 μL of the nanoemulsion labeled either with ^3^ H-cholesteryl oleate and ^14^ C-phosphatidylcholine, or with ^3^ H-triolein and ^14^ C-cholesterol. After a 1-h incubation on a shaker in a water bath at 37°C, 250 μL dextran sulfate/MgCl2 0,2%/MgCl2 3 M, v/v were added as a precipitating reagent. The solution was then mixed for 30 s and centrifuged for 10 min at 3000 g. Finally, 250 μL of the supernatant was added to counting vials containing 5 mL scintillation solution (Packard BioScience, Groningen, Netherlands). Radioactivity was measured with a liquid scintillation analyzer, as described above. The blank samples consisted of 200 μL Tris solution with added labeled nanoemulsion and the precipitation reagent after incubation, as described above. The results of the radioactive transfer from the lipid nanoemulsions to the HDL fractions were expressed as a percentage of the total incubated radioactivity, determined in a plasma sample containing no precipitation reagents. The above described assay was validated for precision according to Food and Drug Administration Guidelines [46] in which the upper limit is 5%. Intra-assay precision studies included 10 replicates of samples. Inter-assay precision was calculated based on 3 assays of 10 replicates, run on 3 different days. Intra-assay coefficient of variation was 0.83% for phospholipids, 0.56% for free cholesterol, 1.49% for esterified cholesterol and 0.51% for triglycerides indicating consistent reproducibility. Inter-assay showed no difference between samples for transfer of all lipids (phospholipids = 0.78; free cholesterol = 0.59; esterified cholesterol = 1.32; triglycerides = 0.58). The assay was performed with maximum plasma storage of two-months after sample collection. As previously tested, the storage of the plasma samples at -80°C up to 1-year had no effect on the lipid transfer results [30].

### Informed consent and radiological safety

The experimental protocol was approved by the Ethics Committee of the Medical School Hospital of the University of São Paulo and all participants provided written informed consent. The safety of the radioactive dose intravenously injected into the patients was assured according to the regulations of the International Commission on Radiological Protection [47]. The injected dose on each experiment was 0.03mSV.

### Statistical analysis

Data normality was tested by the Kolmogorov-Smirnov procedure. Data are expressed as mean ± standard error of the mean. Non- parametric test were performed: Mann–Whitney test, for normally distributed variables and by Fischer Exact test (2- tail), for categorical variable. In all analysis difference of two tail p < 0,05 was considered statically significant. It was used SPSS version 15.0 (IBM Company, Chicago, Illinois, USA) for Windows XP (Microsoft Corporation, Redmond, Washigton, USA) for all the analysis.

## Competing interests

The authors declare that they have no competing interests.

## Authors’ contributions

CPO selected the patients, performed the experiments and data analysis, and wrote the manuscript. RCM conceived of the study, analyzed the results, and wrote the manuscript. MPB performed the experiments and data analysis. BLW designed the clinical protocol, and contributed to discussion. ACL conceived of the study, participated in its design, analyzed the results, and wrote the manuscript. All authors read and approved the final manuscript.
